# The optimal minimum lymph node count for carcinoembryonic antigen elevated colon cancer: a population-based study in the SEER set and External set

**DOI:** 10.1186/s12885-023-10524-y

**Published:** 2023-01-30

**Authors:** Hao Zhang, Chunlin Wang, Yunxiao Liu, Hanqing Hu, Qingchao Tang, Rui Huang, Meng Wang, Guiyu Wang

**Affiliations:** 1 Department of Colorectal Surgery, the Second Affiliated Hospital of Harbin Medical University, 157 Baojian Road, Harbin, Heilongjiang China; 2 Department of Colorectal Cancer Surgery, Cancer Hospital of the University of Chinese Academy of Sciences (Zhejiang Cancer Hospital), 38 Guangji Road, Zhejiang, Hangzhou China

**Keywords:** Colon cancer, CEA-elevated disease, Lymph node, SEER

## Abstract

**Purpose:**

The aim of this paper was to clarify the optimal minimum number of lymph node for CEA-elevated (≥ 5 ng/ml) colon cancer patients.

**Methods:**

Thirteen thousand two hundred thirty-nine patients from the SEER database and 238 patients from the Second Affiliated Hospital of Harbin Medical University (External set) were identified. For cancer-specific survival (CSS), Kaplan-Meier curves were drawn and data were analyzed using log-rank test. Using X-tile software, the optimal cut-off lymph node count was calculated by the maximal Chi-square value method. Cox regression model was applied to perform survival analysis.

**Results:**

In CEA-elevated colon cancer, 18 nodes were defined as the optimal minimum node. The number of lymph node examined (< 12, 12-17 and ≥ 18) was an independent prognosticator in both SEER set (HR_12-17 nodes_ = 1.329, *P* <  0.001; HR_< 12 nodes_ = 1.985, *P* <  0.001) and External set (HR_12-17 nodes_ = 1.774, *P* <  0.032; HR_< 12 nodes_ = 2.741, *P* <  0.006). Moreover, the revised 18-node standard could identify more positive lymph nodes compared with the 12-node standard in this population.

**Conclusions:**

With the purpose of favorable long-term survival and accurate nodal stage for CEA-elevated colon cancer patients, the 18-node standard could be regarded as an alternative to the 12-node standard advocated by the ASCO and NCCN guidelines.

**Supplementary Information:**

The online version contains supplementary material available at 10.1186/s12885-023-10524-y.

## Introduction

Globally, colon cancer constitutes a major public health challenge because of its high incidence and mortality rate [1]. By 2030, the global prevalence rate is estimated to increase by approximately 60%, and colon cancer could be a severe social burden, with more than 1.1 million deaths and 2.2 million new cases [2].

In recent years, clinical schedules for colon cancer become standardized and streamlined. Lymph node count, as a crucial postoperative pathological data, plays an important role in the accurate estimation of patient prognosis and rational formulation of therapeutic scheme [3–5]. Based on the American Society of Clinical Oncology (ASCO) and the National Comprehensive Cancer Network (NCCN) guidelines, a minimum of 12 lymph node counts is essential to ensure proper lymphadenectomy and accurate tumor stage [6]. Although the 12-node standard has been advocated, the literature lacks consensus as to what is the minimal number of lymph nodes to accurately identify stage II cancer and therefore, the proposed standard might be unsuitable for those with node-negative disease.

Carcinoembryonic antigen (CEA) is an irreplaceable tumor marker of colon cancer. In 1965, CEA was reported firstly as a member of the immunoglobulin superfamily [7]. Secreted by various solid tumors, CEA could be found in increased level in 90% of colorectal cancer patients [8]. Moreover, CEA could accelerate tumor progression and support colon cancer cells to attach to the metastatic sites and was associated with unfavorable long-term survival [9–12]. In 2000, the Colorectal Working Group of AJCC even recommended the serum level of CEA should be added into conventional AJCC TNM staging system of colon cancer [6]. Therefore, more attention should be paid in CEA-elevated colon cancer patients due to their distinct characteristics.

Regarding the optimal number of lymph node examination, the level of CEA should also be taken into account. CEA-elevated colon cancer is related to a more aggressive biological property and need a more adequate lymphadenectomy to guarantee the curative resection, and therefore, the conventional 12-node standard might be insufficient for this special population. Hence, the aim of this paper was to recalculate the optimal minimum lymph node count for colon cancer patients with CEA-elevated (≥ 5 ng/ml) disease [13], with data from the Surveillance, Epidemiology, and End Results (SEER) program and the Second Affiliated Hospital of Harbin Medical University.

## Methods

### Study population

Data was extracted from the Surveillance, Epidemiology, and End Results (SEER) program between January 2010 and December 2015 (user ID: 14262 - Nov2019). The National Cancer Institute’s SEER database collects cancer diagnosis, treatment, and survival data for approximately 30% of the U.S. population from 18 participating population-based cancer registries annually. In addition, data from the Second Affiliated Hospital of Harbin Medical University between January 2011 and December 2015 were also included in the current research as an External set.

Inclusion criteria included: (1) radical resection was the first course of treatment; (2) patients with CEA-elevated disease; (3) aged ≥18 years; (4) patients diagnosed as nonmetastatic colon cancer pathologically; (5) colon cancer was the only malignancy. Exclusion criteria included: (1) patients underwent neoadjuvant chemoradiotherapy; (2) patients with unknown race, grade, tumor size, histological type, number of lymph node examined and tumor stage and (3) patients without active follow-up.

In this study, cecum, ascending colon, hepatic flexure and transverse colon were considered as right colon, and splenic flexure, descending colon and sigmoid colon were considered as left colon [14]. Based on the pathological examination of surgical specimens, all cases were uniformly re-staged according to the 8th edition of the American Joint Committee on Cancer (AJCC) tumor-node-metastasis (TNM) staging system.

### Statistical analysis

Patients’ characteristics were demonstrated by number and percentage. For cancer-specific survival (CSS), Kaplan-Meier curves were drawn and data were analyzed using log-rank test. Based on the cancer specific survival, X-tile was used to confirm the relationship between long-term outcome and different lymph node count based on the projection of each possible cut-off point [15]. And then, the optimal cut-off point was calculated by selecting minimum *P* value with the maximum Chi-square value in all possible subdivisions of the populations.

To assess the clinical value of this revised standard, univariate and multivariate Cox regression models were conducted to examine the hazard rate (HR) and the exact 95% confidence interval (CI). ANOVA was utilized to compare the mean positive lymph node count between different subsets. All statistical analyses were performed using SPSS 22.0, and *P* value < 0.05 (two-sided) was considered to be statistical significance.

## Results

### Patient characteristics

Thirteen thousand two hundred thirty-nine patients from the SEER database (SEER set) and 228 patients from the Second Affiliated Hospital of Harbin Medical University (External set) were identified. In SEER set and External set, patients with right colon (62.3% for SEER set, 51.7% for External set), adenocarcinoma (86.4% for SEER set, 89.9% for External set), Grade I/II (78.2% for SEER set, 84.0% for External set), tumor size ≥5 cm (54.6% for SEER set, 52.9% for External set) and AJCC stage I/II (52.1% for SEER set, 62.6% for External set) made up the majority of enrolled cases. In the SEER set, larger proportion was found in female (54.4%), while male (59.2%) occupied the larger proportion in the External set. The number of lymph node examined in the SEER and External sets were 20.1 ± 9.6 and 18.2 ± 7.6, respectively. And the number of positive lymph node examined in the SEER and External sets were 2.0 ± 3.7 and 1.4 ± 3.0, respectively. More detailed data could be found in Table 1. In addition, 26,015 CEA-normal colon cancer patients in the SEER database were also included in the study and to be compared with CEA-elevated patients regarding lymph node count and positive lymph node count, and we found that although there was no significant difference between the two CEA level patients, patients with CEA-elevated colon cancer had higher positive lymph node count compared to those with CEA-normal disease (Fig. S1), which meant CEA-elevated colon cancer were more likely to associated with higher rate of lymph node metastasis, and therefore, requiring more sufficient lymphadenectomy.

**Table 1:** Characteristics of patients in the SEER and External sets

Characteristics	SEER set (***N*** = 13,239)	External set (***N*** = 238)
**Race**		
White	9633 (72.8)	0 (0.0)
Black	2083 (15.7)	0 (0.0)
Other	1523 (11.5)	238 (100.0)
**Gender**		
Male	6028 (45.5)	141 (59.2)
Female	7211 (54.5)	97 (40.8)
**Tumor location**		
Right colon	8246 (62.3)	123 (51.7)
Left colon	4993 (37.7)	115 (48.3)
**Histological type**		
Adenocarcinoma	11,445 (86.4)	214 (89.9)
Mucinous/signet ring-cell	1453 (11.0)	24 (10.1)
Other	341 (2.6)	0 (0.0)
**Grade**		
Grade I/II	10,354 (78.2)	200 (84.0)
Grade III/IV	2885 (21.8)	38 (16.0)
**Tumor size (cm)**		
< 5	6012 (45.4)	112 (47.1)
≥ 5	7227 (54.6)	126 (52.9)
**Age (years)**	68.1 ± 13.9	61.1 ± 12.9
**AJCC stage**		
I/II	6894 (52.1)	149 (62.6)
III	6345 (47.9)	89 (37.4)
**Lymph node count**	20.1 ± 9.6	18.2 ± 7.6
**Positive lymph node count**	2.0 ± 3.7	1.4 ± 3.0

### Optimal minimum lymph node count for CEA-elevated colon cancer patients

**Fig. 1:**
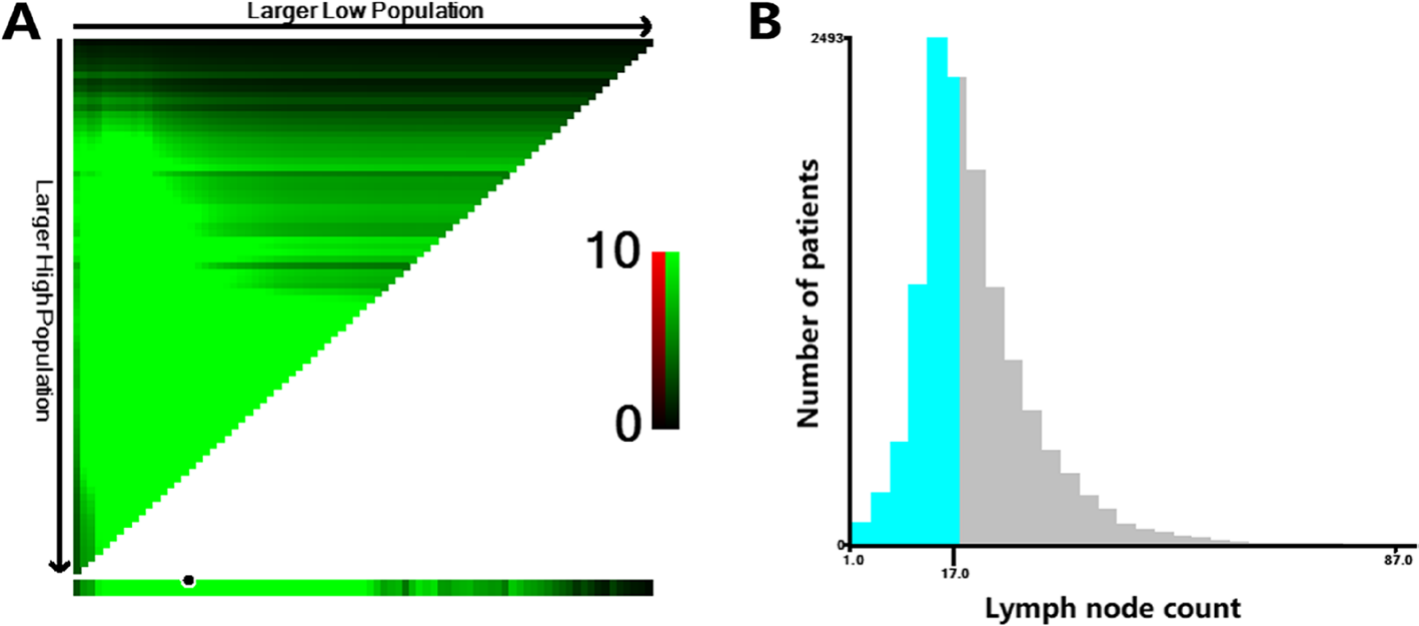
X-tile analysis of CSS in the SEER set. **A** X-tile plots for number of lymph nodes constructed by CEA-elevated patients. The plots show the χ2 log-rank values produced, dividing them into two groups by the cut-off point 18. The brightest pixel represents the maximum χ2 log-rank value. **B** The distribution of number of CEA-elevated patients according to lymph nodes count. Number of lymph nodes ranged from 1 to 87

According to the X-tile program, 18 nodes were defined as the optimal minimum node for CEA-elevated colon cancer patients in the SEER set (< 18 nodes vs. ≥ 18 nodes: log-rank *P* <  0.001), with the maximum Chi-square value 88.5 (Fig. 1). Subsequently, the proposed optimal minimum node count was introduced into further analyses.

### Survival analyses

**Fig. 2:**
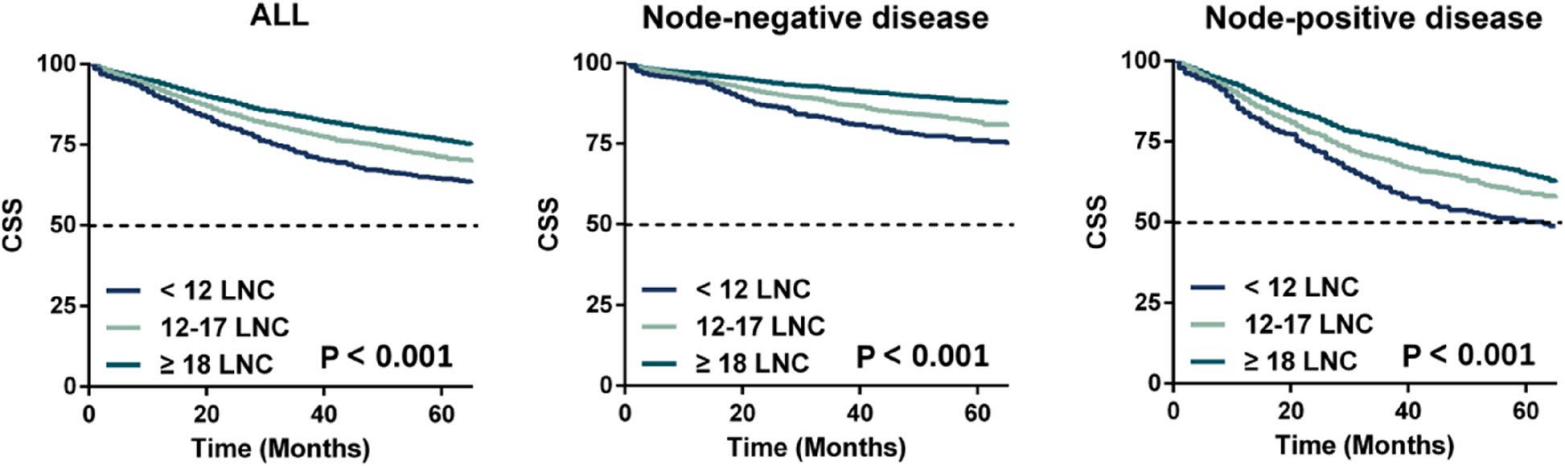
Kaplan–Meier survival curves stratified by number of lymph node examined (< 12 vs. 12–17 vs. ≥ 18 nodes) in the SEER set

**Fig. 3:**
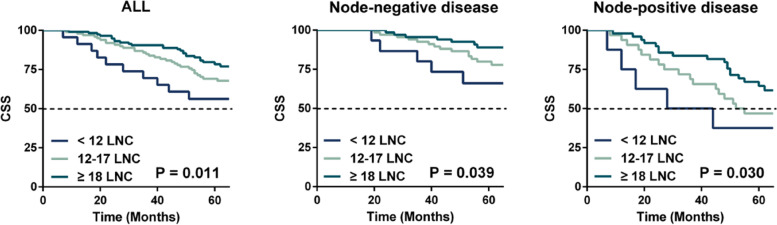
Kaplan–Meier survival curves stratified by number of lymph node examined (< 12 vs. 12–17 vs. ≥ 18 nodes) in the External set

All patients were stratified into three subsets based on the lymph node count (< 12, 12-17 and ≥ 18), and Kaplan-Meier survival curves were plotted in Fig. 2 (SEER set) and Fig. 3 (External set). We could find that patients with lymph node count ≥18 posed better CSS compared with those with lymph node count < 12 and 12-17 in both Stage I/II (*P* <  0.001 for SEER set, *P* = 0.039 for External set) and Stage III (*P* <  0.001 for SEER set, *P* = 0.030 for External set) colon cancer.

What is more, univariate and multivariate Cox model also suggested lymph node count (< 12, 12-17 and ≥ 18) was still a prognostic indicator even after the adjustment for relevant significant variables (In SEER set: HR = 1.329, 95%CI = 1.224-1.444 for 12-17 nodes, *P* <  0.001; HR = 1.985, 95%CI = 1.774-2.221 for < 12 nodes, *P* <  0.001; In External set: HR = 1.774, 95%CI = 1.050-2.998 for 12-17 nodes, *P* = 0.032; HR = 2.741, 95%CI = 1.335-5.627 for < 12 nodes, *P* = 0.006, using ≥18 nodes as the reference) (Tables 2 and 3).

**Table 2:** Cox regression analyses for CSS in the SEER set

Characteristics	Univariate analyses		Multivariate analyses	
	HR [95% CI]	***P***	HR [95% CI]	***P***
**Race**				
White	1		1	
Black	1.005 [0.907-1.114]	0.925	1.102 [0.993-1.223]	0.066
Other	0.881 [0.778-0.998]	0.046	0.876 [0.773-0.993]	0.038
**Gender**				
Male	1			
Female	1.060 [0.983-1.142]	0.132		
**Tumor location**				
Right colon	1		1	
Left colon	0.837 [0.774-0.905]	< 0.001	1.010 [0.929-1.097]	0.820
**Histological type**				
Adenocarcinoma	1		1	
Mucinous/signet ring-cell	1.343 [1.203-1.499]	< 0.001	1.197 [1.070-1.339]	0.002
Other	1.871 [1.550-2.258]	< 0.001	1.415 [1.169-1.713]	< 0.001
**Grade**				
Grade I/II	1		1	
Grade III/IV	1.990 [1.838-2.155]	< 0.001	1.548 [1.424-1.683]	< 0.001
**Tumor size (cm)**				
< 5	1			
≥ 5	1.267 [1.174-1.367]	< 0.001	1.289 [1.192-1.394]	< 0.001
** Age**	1.029 [1.026-1.032]	< 0.001	1.032 [1.029-1.035]	< 0.001
**AJCC stage**				
I/II	1		1	
III	2.610 [2.408-2.828]	< 0.001	2.851 [2.625-3.096]	< 0.001
**Lymph node count**				
≥ 18	1		1	
12-17	1.316 [1.213-1.428]	< 0.001	1.329 [1.224-1.444]	< 0.001
< 12	1.819 [1.630-2.029]	< 0.001	1.985 [1.774-2.221]	< 0.001

**Table 3:** Cox regression analyses for CSS in the External set

Characteristics	Univariate analyses		Multivariate analyses	
	HR [95% CI]	***P***	HR [95% CI]	***P***
**Gender**				
Male	1			
Female	1.020 [0.634-1.643]	0.934		
**Tumor location**				
Right colon	1			
Left colon	1.099 [0.688-1.756]	0.694		
**Histological type**				
Adenocarcinoma	1			
Mucinous/signet ring-cell	1.463 [0.725-2.952]	0.288		
**Grade**				
Grade I/II	1			
Grade III/IV	1.477 [0.822-2.654]	0.192		
**Tumor size (cm)**	1			
< 5	1		1	
≥ 5	1.783 [1.093-2.910]	0.021	1.414 [0.859-2.327]	0.173
**Age**	1.032 [1.011-1.054]	0.002	1.030 [1.008-1.052]	0.006
**AJCC stage**				
I/II	1		1	
III	3.301 [2.038-5.348]	< 0.001	3.506 [2.141-5.742]	< 0.001
**Lymph node count**				
≥ 18	1		1	
12-17	1.546 [0.924-2.587]	0.097	1.774 [1.050-2.998]	0.032
< 12	2.813 [1.389-5.699]	0.004	2.741 [1.335-5.627]	0.006

### The 18-node standard was associated with changed tumor stage and positive lymph node count

Subsequently, the proportions of AJCC stage and N stage and the number of positive lymph node were compared between the three subsets stratified by lymph node count (< 12, 12-17 and ≥ 18 nodes).

**Fig. 4:**
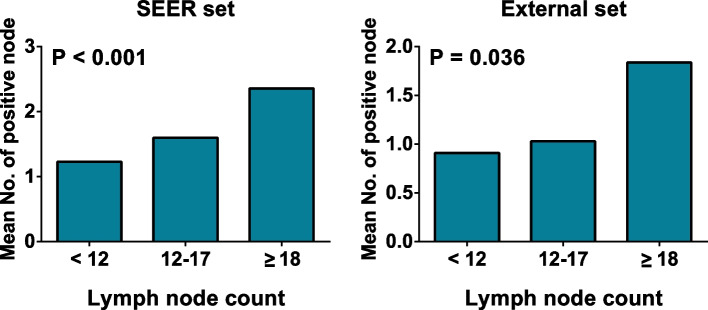
Mean number of positive nodes in different subsets

In the SEER set, with more lymph nodes harvested, the percentage of AJCC stage III (*P* <  0.001) (Table 4) and N2 stage (*P* <  0.001) (Table 5) increased. Besides, the mean positive lymph node count also notably differed between the three subsets and was highest in the ≥18 nodes subsets (Fig. 4).

**Table 4:** Change of AJCC stage in different subsets of lymph nodes examined

Set	AJCC stage	< 12 nodes	12-17 nodes	≥ 18 nodes	***P***
**SEER set (*N*** **= 13,239)**	I/II	782 (55.0)	2593 (53.8)	3519 (50.3)	< 0.001
	III	640 (45.0)	2229 (46.2)	3476 (49.7)	
**External set (*N*** **= 238)**	I/II	15 (65.2)	67 (67.7)	67 (57.8)	0.314
	III	8 (34.8)	32 (32.3)	49 (42.2)

**Table 5:** Change of N stage in different subsets of lymph nodes examined

Set	N stage	< 12 nodes	12-17 nodes	≥ 18 nodes	***P***
**SEER set (*N*** **= 13,239)**	N0	782 (55.0)	2593 (53.8)	3519 (50.3)	< 0.001
	N1	459 (32.3)	1404 (29.1)	1976 (28.2)	
	N2	181 (12.7)	825 (17.1)	1500 (21.5)	
**External set (*N*** **= 238)**	N0	15 (65.2)	67 (67.7)	67 (57.8)	0.048
	N1	7 (30.5)	25 (25.3)	26 (22.4)	
	N2	1 (4.3)	7 (7.0)	23 (19.4)

In the External set, there was no difference in AJCC stage with the lymph node count increasing (*P* = 0.314) (Table 4). However, the proportion of N2 stage significantly changed in ≥18 nodes subsets (*P* = 0.048) (Table 5), but not 12-17 nodes subsets. And compared with the other two subsets, the number of positive lymph node examined was also obviously higher in ≥18 nodes subsets (*P* = 0.036) (Fig. 4). Therefore, compared to conventional 12-node standard, at least examining 18 nodes was linked to more positive lymph node examined and accurate tumor stage.

## Discussion

CEA is a recommended prognostic marker for monitoring tumor progression in colon cancer [16–19], and examining 12 nodes might be insufficient for CEA-elevated colon cancer patients who were characterized by higher risk of lymph node metastasis and poor prognosis. In this paper, we found that 18-node standard could be deemed as an alternative to the conventional 12-node standard, as a result of the accurate nodal stage and favorable prognosis.

In recent years, many scholars have reassessed the optimal lymph node yield for colon cancer patients according to different stratifications. With the consideration of anatomic factor [20], Guan et al. revealed that at least 15 lymph nodes examined in patients with stage I-III right colon cancer could significantly improve the 5-year CSS and the rate of node-positive disease [21]. Similarly, Cai et al. concluded that a minimum of 19 lymph nodes harvested is essential in stage II right colon cancer, with the aim of favorable survival [18]. Regarding early-on set colon cancer cases, Guan also suggested that 22-node standard should be recommended, which required more lymph nodes than conventional guidelines [16]. And for N0 colon cancer, Ning et al. thought that harvesting at least 18 nodes was related to better postoperative survival than the 12-node measure [17]. However, few papers have determined the minimum optimal lymph node count for CEA-elevated patients.

Compared with above researches, the merits of current paper were twofold. On the one hand, this was the first study to explore the optimal minimum node for CEA-elevated cases, with the consideration of nodal stage and postoperative prognosis, which was consistent with the studies mentioned above. On the other hand, different from the above articles, the results in this study were also validated using an External set, which suggested the proposed standard might also apply to Chinese population, making it more convincing than others.

To our knowledge, the potential mechanisms accounting for the improved survival of patients with increased lymph node yield were multifactorial. Firstly, increased lymph node count was related to a more robust antitumor immune response, a significant indicator of favorable prognosis [22]. Patients with less lymph nodes retrieved might have lowered resistance to metastasis and recurrence, therefore occupying decreased survival time. Secondly, lymph node count was a surrogate marker to evaluate the performance of radical surgery. According to previous studies, surgeons’ skills in lymph nodes dissection is of vital significance for patients’ survival [23]. Patients with higher number of lymph node retrieved were more likely to accept an adequate radical cure, leading to the presence of improved survival. Thirdly, in this paper, we found the number of lymph nodes harvested was associated with nodal stage and the number of positive lymph node examined, and therefore, higher lymph node count could reduce the likelihood of false staging, making more patients benefit from adjuvant chemotherapy, which is of eminent significance for their favorable long-term survival.

Findings of the research present eminent significance in clinical work. On the one hand, since more lymph node examination is crucial for CEA-elevated patients, lymphatic tracer such as methylene blue and carbon nanoparticles suspension could be applied for this population to make the lymph node more phanerous for pathologists and surgeons [24]. On the other hand, in the survival evaluation for CEA-elevated cancer, < 18 lymph node count might be a risk factor should be taken into consideration to better perform prognostic stratification. However, the proposed result could only provide a potential reference, but not guidance.

Still, the authors acknowledged several study limitations. Firstly, SEER database collects information about 28% of the U.S. population, leading to dramatic changes in surgical and pathological techniques used to detect lymph nodes and it could not be adjusted in this paper. Secondly, as a retrospective study, it was inevitable to have observer and confusion bias and needed to be verified by some prospective clinical studies. Thirdly, the sample size of the External set seems to be insufficient and we would like to conduct a larger scale study to further authenticate the performance of the 18-node standard in CEA-elevated colon cancer patients. Finally, some potential prognosticators such as the molecular markers and detailed information about surgical procedures are not available in the SEER database and could not be included in the survival analysis.

## Conclusion

In the present study, 18 nodes were determined as the optimal minimum nodes for CEA-elevated colon cancer patients. Examining at least 18 lymph nodes could decrease the risk of nodal understaging and maximize prognostic benefit from lymphadenectomy, and therefore, should be deemed as an alternative to the 12-node standard in this patient population.

## Supplementary Information

**Additional file 1.**

## Data Availability

Data from SEER database are available in the Surveillance, Epidemiology, and End Results cancer registry (https://seer.cancer.gov) and can also be provided by the corresponding author.
