# Supplementary material for: The optimal minimum lymph node count for carcinoembryonic antigen elevated colon cancer: a population-based study in the SEER set and External set
Source: BMC Cancer. 2023 Jan 30;23:100. doi: 10.1186/s12885-023-10524-y (PMC9885584; doi:10.1186/s12885-023-10524-y)

**Fig. S1** Mean number of total and positive nodes in CEA-normal and CEA-elevated colon cancer in the SEER set.


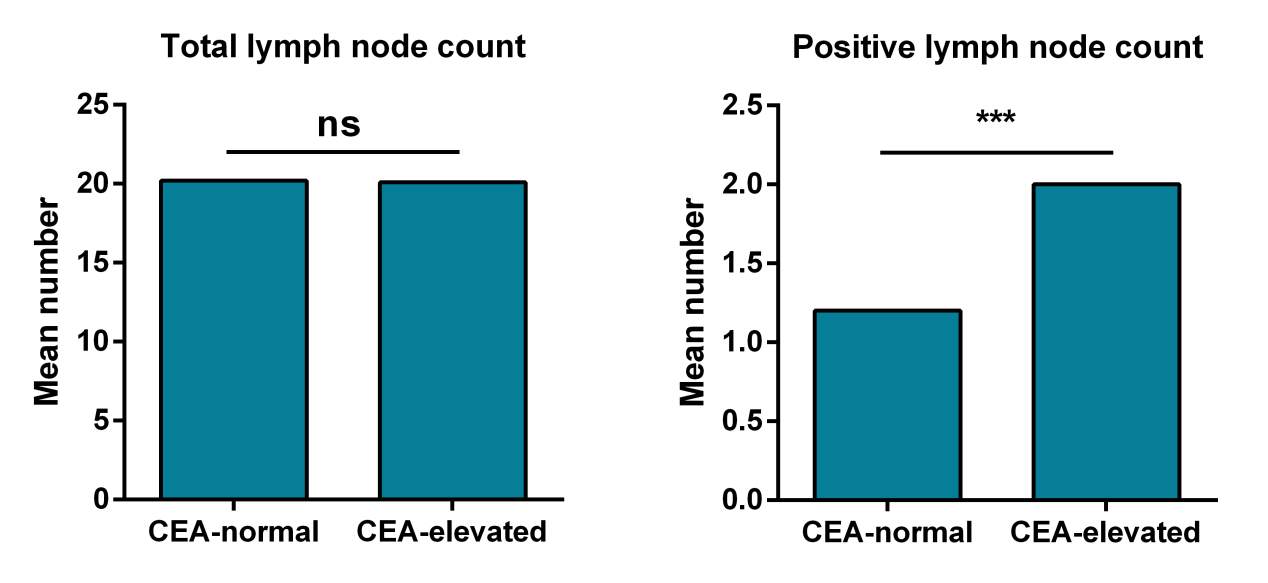

Supplement: Supplementary file 1 — Additional file 1. [file 12885_2023_10524_MOESM1_ESM.docx]
